# Testing the therapeutic effects of transcranial direct current stimulation (tDCS) in semantic dementia: a double blind, sham controlled, randomized clinical trial

**DOI:** 10.1186/s13063-019-3613-z

**Published:** 2019-11-20

**Authors:** Clara Sanches, Richard Levy, Sarah Benisty, Lisette Volpe-Gillot, Marie-Odile Habert, Aurelie Kas, Sébastian Ströer, Nadya Pyatigorskaya, Anna Kaglik, Angelina Bourbon, Bruno Dubois, Raffaella Migliaccio, Antoni Valero-Cabré, Marc Teichmann

**Affiliations:** 1 Institut du Cerveau et de la Moelle Epinière, ICM, INSERM U 1127, CNRS UMR 7225, Sorbonne Université, Frontlab team, Paris, France; 2 Groupe de Dynamiques Cérébrales, Plasticité et Rééducation, FrontLab team, Institut du Cerveau et de la Moelle Epinière, ICM, INSERM U 1127, CNRS UMR 7225, Sorbonne Université, Paris, France; 3 Department of Neurology, National Reference Center for « Rare or Early Onset Dementias », Pitié Salpêtrière Hospital, AP-HP, 47-83 Boulevard de l’Hôpital, 75013 Paris, France; 4 Fondation Ophtalmologique Rothschild, Paris, France; 5 Espace IMAGINE, Hôpital Léopold Bellan, Paris, France; 6 Department of Nuclear Medicine, Hôpital de la Pitié-Salpêtrière, AP-HP, Paris, France; 7 CATI Multicenter Neuroimaging Platform, Paris, France; 8 Laboratoire d’Imagerie Biomédicale, Sorbonne Université, Inserm U1146, CNRS UMR, Paris, France; 9 Department of Neuroradiology, Hôpital de la Pitié-Salpêtrière, AP-HP, Paris, France; 10 Institut du Cerveau et de la Moelle Epinière, Center for NeuroImaging Research – CENIR, Paris, France; 11 Unité de Recherche Clinique (URC) Pitié-Salpêtrière, Charles Foix, AP-HP, Paris, France; 12 Laboratory for Cerebral Dynamics Plasticity and Rehabilitation, Boston University School of Medicine, Boston, MA USA; 13 Cognitive Neuroscience and Information Technology Research Program, Open University of Catalonia (UOC), Barcelona, Spain

**Keywords:** Non-invasive brain stimulation, Transcranial direct current stimulation, Semantic dementia, Primary progressive aphasia, Language impairments, Neurodegenerative diseases, Neurology

## Abstract

**Background:**

Semantic dementia is a neurodegenerative disease that primarily affects the left anterior temporal lobe, resulting in a gradual loss of conceptual knowledge. There is currently no validated treatment. Transcranial stimulation has provided evidence for long-lasting language effects presumably linked to stimulation-induced neuroplasticity in post-stroke aphasia. However, studies evaluating its effects in neurodegenerative diseases such as semantic dementia are still rare and evidence from double-blind, prospective, therapeutic trials is required.

**Objective:**

The primary objective of the present clinical trial (STIM-SD) is to evaluate the therapeutic efficacy of a multiday transcranial direct current stimulation (tDCS) regime on language impairment in patients with semantic dementia. The study also explores the time course of potential tDCS-driven improvements and uses imaging biomarkers that could reflect stimulation-induced neuroplasticity.

**Methods:**

This is a double-blind, sham-controlled, randomized study using transcranial Direct Current Stimulation (tDCS) applied daily for 10 days, and language/semantic and imaging assessments at four time points: baseline, 3 days, 2 weeks and 4 months after 10 stimulation sessions. Language/semantic assessments will be carried out at these same 4 time points. Fluorodeoxyglucose positron emission tomography (FDG-PET), resting-state functional magnetic resonance imaging (rs-fMRI), T1-weighted images and white matter diffusion tensor imaging (DTI) will be applied at baseline and at the 2-week time point. According to the principle of inter-hemispheric inhibition between left (language-related) and right homotopic regions we will use two stimulation modalities - left-anodal and right-cathodal tDCS over the anterior temporal lobes. Accordingly, the patient population (*n* = 60) will be subdivided into three subgroups: left-anodal tDCS (*n* = 20), right-cathodal tDCS (*n* = 20) and sham tDCS (*n* = 20). The stimulation will be sustained for 20 min at an intensity of 1.59 mA. It will be delivered through 25cm^2^-round stimulation electrodes (current density of 0.06 mA/cm^2^) placed over the left and right anterior temporal lobes for anodal and cathodal stimulation, respectively. A group of healthy participants (*n* = 20) matched by age, gender and education will also be recruited and tested to provide normative values for the language/semantic tasks and imaging measures.

**Discussion:**

The aim of this study is to assess the efficacy of tDCS for language/semantic disorders in semantic dementia. A potential treatment would be easily applicable, inexpensive, and renewable when therapeutic effects disappear due to disease progression.

**Trial registration:**

ClinicalTrials.gov NCT03481933. Registered on March 2018.

**Electronic supplementary material:**

The online version of this article (10.1186/s13063-019-3613-z) contains supplementary material, which is available to authorized users.

## Introduction

Semantic dementia (SD), also referred to as the semantic variant of primary progressive aphasia (sv-PPA) [1], is part of the spectrum of frontotemporal lobar degeneration and constitutes one of the major clinical variants of this disorder [2]. The onset age of SD is frequently before 65 years [3] and it severely affects the ability to communicate, which generates a major impact on the family and socio-professional life of patients.

SD is characterized by a gradual and severe loss of conceptual knowledge, resulting in anomia, impaired word comprehension and speech that is fluent but empty of content [2], leaving grammar and speech articulation preserved [4]. Although the most prominent deficits concern word meaning [1, 4], SD might eventually cause deterioration of knowledge for all kinds of semantic concepts [5, 6] impacting on face recognition [7], object feature attribution [8], sound-picture matching [9] and object-use [10]. The damage to multi-modal semantic representations, besides the verbal domain, gave birth to the concept of semantic dementia [11]. It therefore appears that sv-PPA is a purely linguistic variant of SD [12] and/or that SD results from the evolving disease course of sv-PPA [13, 14].

At the anatomical level SD affects the anterior temporal lobe (ATL) of both hemispheres, predominantly in left lateralized regions [1, 4]. Correlation has been identified between gray matter loss in the ATL and different semantic tasks like picture naming [15, 16] and word-picture association [17]. It is associated with disruptions of functional connectivity between a broad range of brain regions across the temporal, frontal, parietal and occipital lobes, including visual and auditory association cortices [18]. Alterations of structural connectivity such as a white matter volume reduction in the left temporal lobe, the periventricular white matter and the corpus callosum [19], and damage to white matter tracts such as the inferior longitudinal fasciculus (ILF) and the uncinate fasciculus (UF) have also been found in SD [20]. Signs of cortical hypometabolism, which are a useful neuroimaging hallmark for diagnosis have mainly been found in the ATL cortices, sometimes extending to the subgenual region and the right anterior cingulate cortex [21].

There is currently no validated treatment for SD given that speech therapy protocols have not been validated and pharmacological trials did not demonstrate significant effects [22–24]. In this context, new approaches based on the use of non-invasive brain stimulation and neuro-modulation [25] might represent a promising therapeutic strategy. Two of the most common technologies for non-invasive brain stimulation are repetitive transcranial magnetic stimulation (TMS) and transcranial direct current stimulation (tDCS) [26]. Repetitive TMS involves the application of a series of magnetic pulses through a stimulating coil placed in contact with an area of the scalp. In a frequency-dependent and pattern-dependent manner, this stimulation technology induces an intracranial electric current that subsequently modulates (inhibits or facilitates) neuronal activity [26]. Repetitive TMS is characterized by its excellent spatial and temporal resolution and ability to make neurons discharge, features that come at the cost of low portability, high financial cost and epileptic risk. The effects of tDCS are based on a weak electric current (1–2 mA) conveyed between two electrodes (an active and a return) placed on separate locations of the scalp, with the ability to generate a polarization gradient across a large cortical area between electrodes, hence modulating cortical excitability within its boundaries [27]. Two modalities of tDCS are commonly used: Anodal and Cathodal stimulation. Anodal stimulation (in which the anode is placed on the targeted region) shifts the membrane resting potential of local neurons bringing it closer to their firing threshold (i.e. making it more positive hence depolarizing local cells). This modality increases regional excitability and facilitates the activation of neurons hence the production of action potentials in response to physiological inputs (excitatory effects). Cathodal stimulation (in which the cathode is placed on the targeted region), shifts the resting membrane potential of nearby neurons away from their firing threshold (i.e., making it more negative, hence hyperpolarizing local cells). Consequently, this modality decreases regional excitability hindering the likelihood of such neurons to generate an action potential in response to a physiological input (inhibitory effects).

For several years a growing number of studies have explored the effects of non-invasive brain stimulation in patients with aphasia following left hemisphere strokes. These studies rely on the assumption that weak electrical currents can interact with neural networks subtending language and promote neural plasticity, allowing short-term modulations, and eventually clinical recovery. Within these language-related networks, left and right homotopic regions are connected via transcallosal connections [28, 29] which, according to the principle of inter-hemispheric inhibition, tend to convey mutual net inhibitory influences [30]. The use of non-invasive brain stimulation in left hemisphere stroke aphasia relies on three potential mechanisms or combinations thereof [31, 32]: (1) the use of left-excitatory (anodal tDCS or high-frequency TMS) stimulation on left hemisphere language systems to reactivate language processes implemented by peri-lesional regions; (2) the delivery of right-inhibitory (cathodal tDCS or low-frequency TMS) stimulation to reduce the inhibition that right hemisphere systems exert on the left dominant language network and (3) the delivery of right-excitatory stimulation to activate potential language contributions of right hemisphere networks. The most promising clinical outcomes on language disabilities have been achieved from studies adopting the first two approaches using either TMS (e.g., see [33–36]) or tDCS (e.g., see [37–39]). Beneficial effects have been shown either transiently with single-session applications, or as longer-lasting impacts (> 6 months) following periodical stimulation sessions across several days, probably related to stimulation-induced neuroplasticity [34, 40].

Non-invasive brain stimulation has also been used with relative efficacy in Alzheimer’s disease by targeting the left and/or right dorsolateral prefrontal cortex [41, 42]. In PPA, including SD (or sv-PPA), two small-cohort studies have suggested encouraging results with both TMS and tDCS [43, 44]. However, the authors did not target language-specific brain regions and the small number of patients precluded a counterbalanced study design. In addition, left-excitatory versus right-inhibitory TMS or tDCS have not been systematically evaluated to reveal the most efficient strategy in aphasia of neurodegenerative origin.

The purpose of this article is to present a clinical protocol (PHRC “STIM-SD”*)* implementing a multi-day tDCS regime in a large population of patients with SD (*n* = 60) specifically targeting the left ATL (with anodal stimulation) or the right ATL (with cathodal stimulation) to provide evidence for potential therapeutic effects and brain plasticity outlasting the duration of the treatment. This intervention builds on a previous pre-therapeutic double-blind, sham-controlled study by our team using a single tDCS session applied to the left and right ATL in patients with SD [45]. This approach allowed the comparison of left anodal (excitatory) to right cathodal (inhibitory) tDCS stimulation and showed that a single session of both left anodal and right cathodal tDCS resulted in transient but highly significant intra-semantic effects [45]. The goals of the present study include the evaluation of the potential therapeutic efficacy of periodical sessions of tDCS over the ATL during 10 days in language/semantic performance in patients with SD, the assessment of the time course of potential improvements, the exploration of potential effects on brain plasticity using functional connectivity measures (resting-state functional magnetic resonance imaging (fMRI)) and cortical metabolism (fluorodeoxyglucose positron emission tomography (FDG-PET)), the identification of the most efficient stimulation modality (left-anodal versus right-cathodal) and the identification of biomarkers such as ATL atrophy levels, which could be individually indicative of an efficient tDCS impact.

## Methods

### Study design

**Fig. 1:**
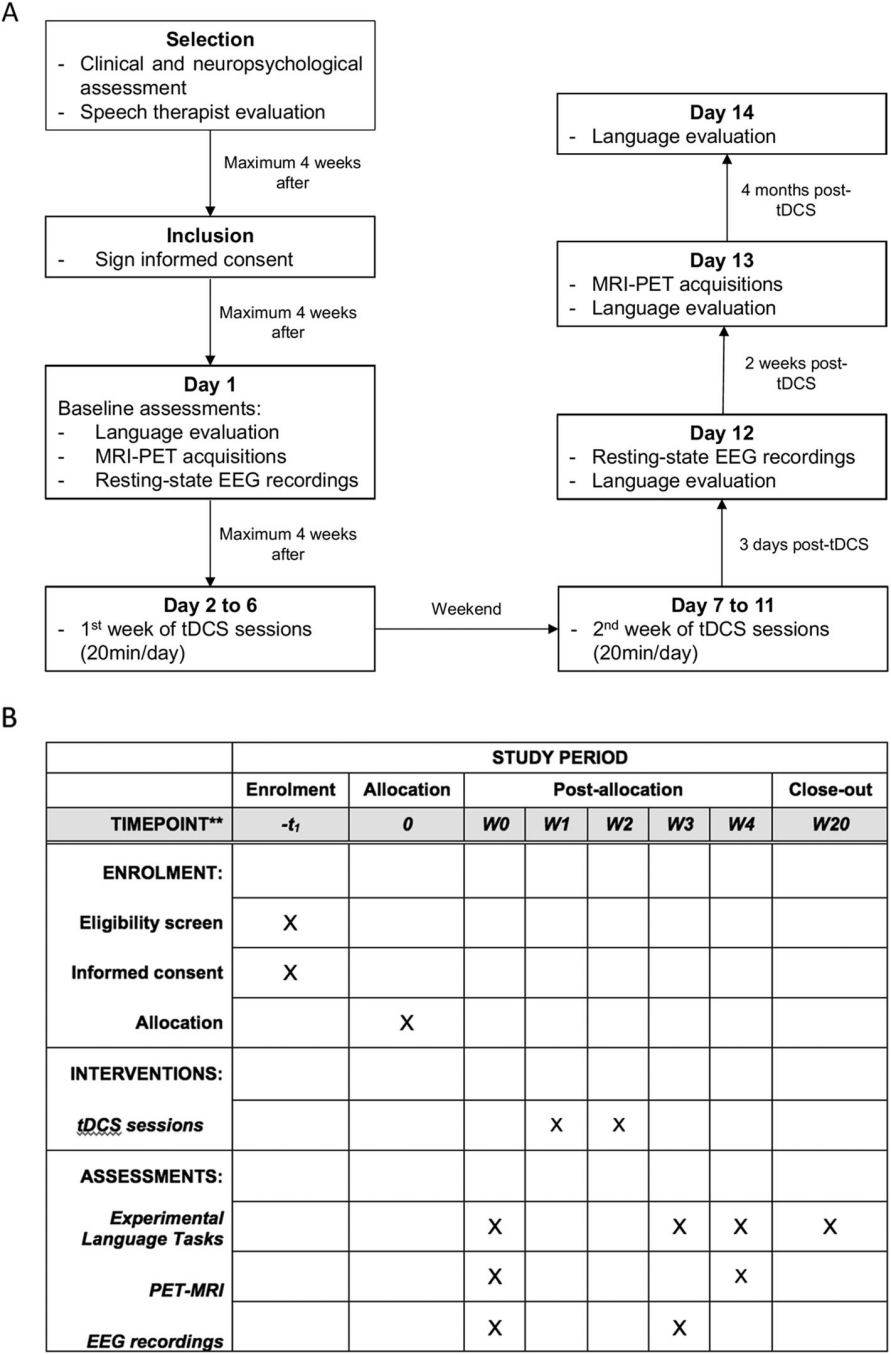
**a** Flow diagram from patients’ selection to the end of their participation in the study. tDCS, transcranial direct current stimulation; MRI, magnetic resonance imaging; PET, positron emission tomography; EEG, electroencephalography. **b** Standard protocol items: recommendations for Interventional Trials (Additional file 1). Table defining the different evaluations or interventions (left column) that would be performed for each study period or time point (top rows)

The *STIM-SD* protocol is a double-blind, sham-controlled, randomized study testing the efficiency of periodical tDCS sessions for 10 days (Monday to Friday for 2 weeks) using language/semantic assessments, and PET and MRI-based neuroimaging at four time points: baseline and 3 days, 2 weeks and 4 months after the end of the tDCS sessions (Fig. 1a, b, and Additional file 1). The patient population (*n* = 60) is randomly assigned to three subgroups each receiving a different treatment over the ATL: left-anodal tDCS (*n* = 20), right-cathodal tDCS (*n* = 20) and sham tDCS (*n* = 20).

Language/semantic assessments are applied at the four time points by a set of computer-based tasks. For each task, we have developed two equivalent versions matched on various linguistic variables, which are used alternatively either at baseline or during follow-up evaluations, in a counterbalanced order across patient subgroups to avoid test/re-test confounds. Neuroimaging acquisitions (structural MRI, resting-state fMRI, FDG-PET) are performed at baseline and the 2-week time point. We also acquire resting-state electroencephalogram (EEG) recordings at baseline and after the end of tDCS sessions (3-day time point) given that patients with SD might have an altered pattern of resting state neuronal synchronizations [46].

To ensure double-blinding a first investigator (stimulator) performs and supervises the tDCS sessions, whereas a second investigator (evaluator) conducts the langue/semantic tasks, blinded to the stimulation condition (anodal, cathodal or sham). During sham stimulation, tDCS current is ramped up and down along 30 s respectively during the initial and final phases of the session, to emulate the transient skin-itching sensations characterizing active anodal or cathodal stimulation. Unnoticed by the patients, the stimulation unit is turned off during the 20-min sham tDCS session.

Stimulation sessions are applied daily for 10 days (Monday to Friday for 2 weeks). Each tDCS session lasts for 20 min. The direct current has an intensity of 1.59 mA (25cm^2^-round electrodes, current density of 0.06 mA/cm^2^). A group of 20 healthy subjects are also evaluated at baseline to provide normative values for language/semantic tasks and for imaging measures. Healthy participants do not undergo tDCS treatment and are only assessed once with the same language/semantic tasks and neuroimaging assessments performed by SD patients.

The primary endpoint of the *STIM-SD* protocol is to evaluate the potential therapeutic efficacy of multiday tDCS (10 days) on language/semantic performance in patients with SD (see the section “Language/semantic tasks” – the semantic association task). In addition, we will also (1) assess the time course of potential language/semantic improvement through the application of four follow-up time points; (2) assess neuroimaging biomarkers (PET and MRI) that could reflect stimulation-induced neuroplasticity and response to stimulation; (3) compare the effects of left-anodal and right-cathodal tDCS to define the most efficient stimulation modality; (4) identify biomarkers that could individually predict tDCS impact and (5) improve the understanding of the semantic roles of the left and right ATL and their potential structural connectivity, contributing to the definition of anatomic-functional models of semantics.

The protocol has been approved by the local Ethics Committee and is registered in ClinicalTrials.gov with the identifier NCT03481933 and the study title “‘Evaluation of a Transcranial Stimulation with Direct Current on Language Disorders in Semantic Dementia (STIM-SD)”*.* Written informed consent is obtained from all patients and healthy subjects before the onset of any of the study procedures. All research protocol visits take place at the same site, the Pitié-Salpêtrière Hospital. Two additional centers contribute to the protocol by recruiting patients with SD.

### Participants

Patients with SD are recruited in the National Reference Center for Rare or Early Onset Dementias at the Pitié-Salpêtrière Hospital, at the Rothschild Ophthalmologic Foundation and at the Léopold Bellan Hospital in Paris. Patients are recruited based on the following inclusion criteria: (1) diagnosis of SD based on current research criteria [1] comprising progressive language impairment, single-word comprehension deficits and anomia, without sentence repetition impairment, agrammatism or motor speech disorders; (2) age > 18 years and (3) affiliation to a social security regime.

Non-inclusion criteria are the following: (1) psychiatric disorders or neurologic diseases other than SD; (2) contraindication for MRI, PET or tDCS such as presence of intracranial ferromagnetic devices, scalp or skull lesions or epilepsy; (3) MRI recordings revealing pathological processes other than those associated with SD; (4) severe aphasia (severity score < 3 in the Boston Diagnostic Aphasia Evaluation (BDAE) [47]); (5) Mini Mental State Examination (MMSE) [48] score < 15; (6) Frontal Assessment Battery (FAB) [49] score < 10; (7) Montgomery Asberg Depression Rating Scale [50] (MADRS) score ≥ 20, indicating a major depressive disorder; (8) not having the French language as a mother tongue; (9) being left-handed and (10) being under curatorship or tutorship. Prior to inclusion in the protocol all patients undergo a neuropsychological and speech therapist evaluation to check for inclusion/non-inclusion criteria, and to characterize global cognitive/language/semantic capacities on the basis of several published standard tests.

Healthy participants are recruited among hospital staff, caregivers and patients’ relatives via announcements in the neurology department. They are matched with the patients with SD on sex, age, handedness and number of years of education. Non-inclusion criteria for healthy participants are the following: (1) neurological or psychiatric disorders or physical deficits that can interfere with cognitive function; (2) contraindications to MRI or PET and (3) not having French language as mother tongue.

### Randomization

A computer-generated block randomization list has been prepared by the Clinical Research Unit of the Pitié-Salpêtrière - Charles Foix Hospital group. The randomization list is integrated into the electronic case report form (eCRF) and a randomization number assigning patients to one of the three stimulation conditions (anodal, cathodal or sham tDCS) is attributed automatically upon completion of inclusion/non-inclusion criteria. The randomization is not stratified by center of inclusion because all stimulation sessions and time-point evaluations are performed at the Pitié Salpêtrière Hospital.

### Stimulation administration

**Fig. 2:**
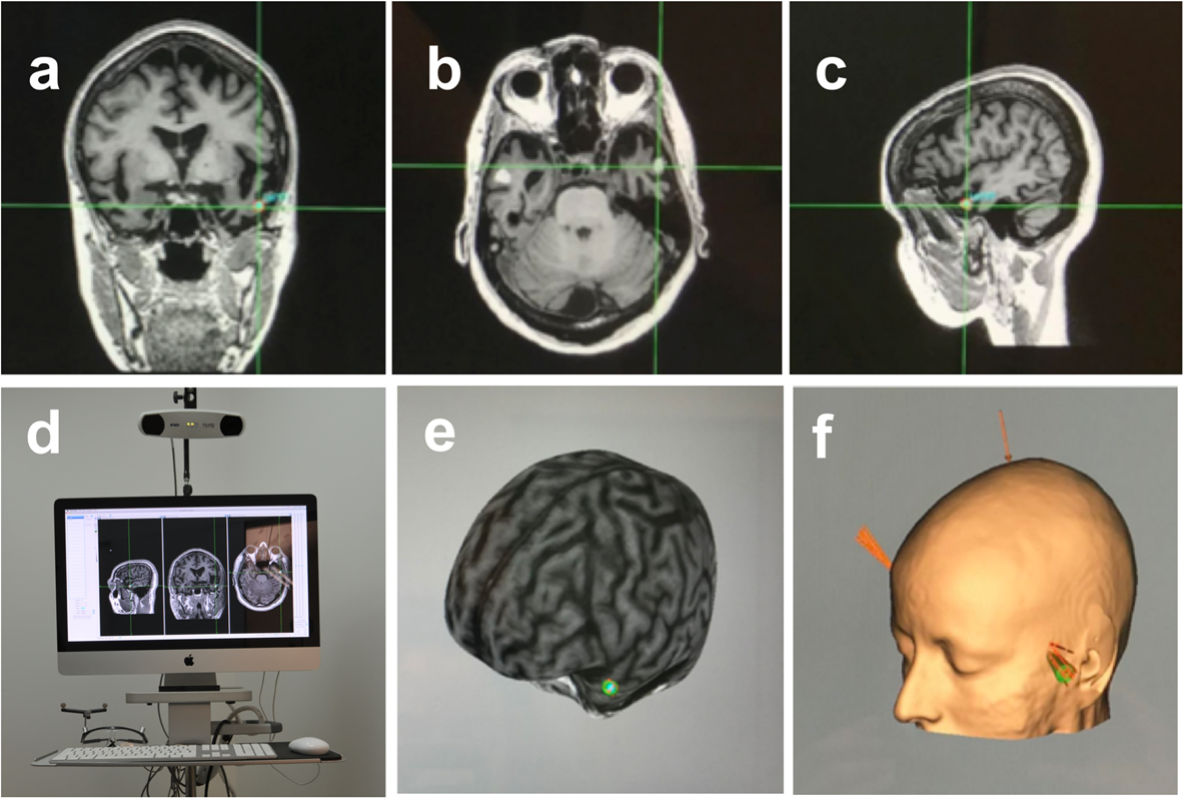
The three upper panels show, respectively, **a** coronal, **b** axial and **c** sagittal sections from magnetic resonance imaging (MRI) in a representative patient with semantic dementia (SD). The crosshair signals the location of the stimulation target in the left Anterior Temporal Lobe (ATL). **d** MRI-based frameless stereotactic navigation system device (Brainsight) employed for accurate targeting of the left ATL in SD patients. 3D brain curvilinear **e** cortical and **f** skin reconstructions from the MRI of a patient, provided by the neuronavigation system with a sphere indicating the left ATL target aimed by the stimulation

The stimulation procedure (electrode montage and stimulation parameters) is the same as the procedure used in our aforementioned pre-therapeutic study [45]. During the baseline visit patients undergo FDG-PET and MRI including anatomical 3D T1-weighted images. The T1-weighted images are registered in standardized Montreal Neurological Institute (MNI) space and the left and right ATL are identified and labeled with a 5-mm sphere centered on MNI coordinates (x = − 52, y = 2, z = − 28) and (x = 53, y = 4, z = − 32), respectively [51], using custom-made SPM8 (Statistical Parametric Mapping, Matlab Mathworks) procedure. Images are then denormalized in each patient’s native space. The day of the stimulation a scalp inspection is performed to verify the absence of skin lesions. Before the placement of the tDCS electrodes the scalp is carefully cleaned using an abrasive paste to limit impedance losses between the skin and the electrodes. Two round sponge electrodes, one acting as the active electrode (anodal or cathode) and the other as return (Sponstim®, 5.65 cm diameter, 25cm^2^ surface, NEuroelectrics, Barcelona, Spain) are placed under MRI guidance using a stereotactic neuronavigation system (Brainsight®, Rogue System®, Montreal, Canada). This procedure minimizes the distance between the labeled cortical target on a 3D reconstruction of each patient’s MR image and its closest (shortest Euclidian path) scalp location (Fig. 2).

Stimulation is delivered using a wireless hybrid EEG/tDCS 8-channel neurostimulator (Starstim, NEuroelectrics, Barcelona, Spain). The active electrode is placed over the left ATL for left-anodal tDCS (between 10 and 20 EEG coordinates ~ FT7 and FT9), while the cathodal tDCS targets the right ATL (between ~ FT8 and FT10). In sham tDCS stimulation, the active electrode is placed over the same MNI coordinates as left-anodal tDCS. The return electrode is placed over the contralateral supra-orbital region with regards to the active electrode location (10–20 EEG coordinates AF8 for left anodal tDCS and AF7 for right cathodal tDCS). Additionally, six EEG scalp electrodes (NG Geltrode® Ag/AgCl, 1.0 cm^2^) provided by the same tDCS device, which allow for continuous monitoring of brain activity during the session, are placed in 10–20 EEG system locations F4, F3, C4, C3 and P4, P3.

During anodal or cathodal tDCS, current intensity is linearly increased over 30 s to reach a maximum of 1.59 mA. This level of tDCS intensity has been chosen to ensure similar levels of current density (0.06 mA/cm^2^) with our 25cm^2^ electrodes as those applied in previous post-stroke aphasia or PPA studies with larger leads [37, 45, 52]. Current is kept at this intensity for 20 min before being ramped down along 30 s at the end of the tDCS session. During sham stimulation the tDCS current is ramped up and down along 30 s at the initial and final phase of the session but is turned off during the 20 min session. This process makes active (anodal or cathodal) and sham stimulations similar (same somatosensory skin sensations) as required by a double-blind design. During each stimulation session, values of mean voltage (V), mean current intensity (uA) and mean impedance (Ohm), directly provided by the stimulation software, are recorded for means of tDCS verification intents. Treatment duration has been chosen based on previous studies showing beneficial tDCS effects with the same amount of time (e.g., [44, 45]). During tDCS sessions patients perform a language-neutral visuo-motor task on a laptop screen consisting of pressing the space bar on a computer keyboard every time a slowly moving dot contacts the edge of a surrounding rectangle. This task is intended to limit variability in neural activity states across sessions and patients (forcing patients to maintain vigilance) without interfering with language processes and tasks. At each session, performances (number of trials, number of successes and number of errors) in the visuo-motor task are recorded.

To ensure safety and comfort and to assess the tolerance of patients to stimulation, immediately after each tDCS session patients are asked to complete a tDCS adverse effects questionnaire [53] that measures, through a rating scale, patients’ sensations in a set of the most frequent adverse effects reported in tDCS studies such as itching, tingling, burning sensations, skin redness or sleepiness.

### Language/semantic tasks

A set of computer-based language/semantic tasks is used to assess the potential effectiveness of tDCS. These tasks are carried out by patients at four time points: pre-tDCS (baseline) and 3 days, 2 weeks and 4 months after the end of the 10-day tDCS regime. The five language/semantic tasks are the following:

- Semantic association task (SA)
- Picture naming task (NAME)
- Reading task (READ)
- Letter and category fluency task (FLU)
- Category judgment task (CJ)

Two additional tasks evaluating tDCS impacts on other cognitive functions are also applied:

- Executive function task (EXE)
- Recognition of famous faces - a subtest of the French *Batterie Imagerie-Perception* (BIP).

The tasks are computer-programmed using E-Prime software (E-Prime®, Psychology Software Tools, Sharpsburg, PA, USA). Participants are comfortably seated in front of a laptop computer screen (HP EliteBook 8770w, USA) and a response box that records responses. Performance accuracy, reaction times and voice records are automatically registered by the software. The sessions are carried out in the presence of an investigator immediately after familiarization/training blocks comprising five trials for each task.

#### Semantic association task (SA)

**Fig. 3:**
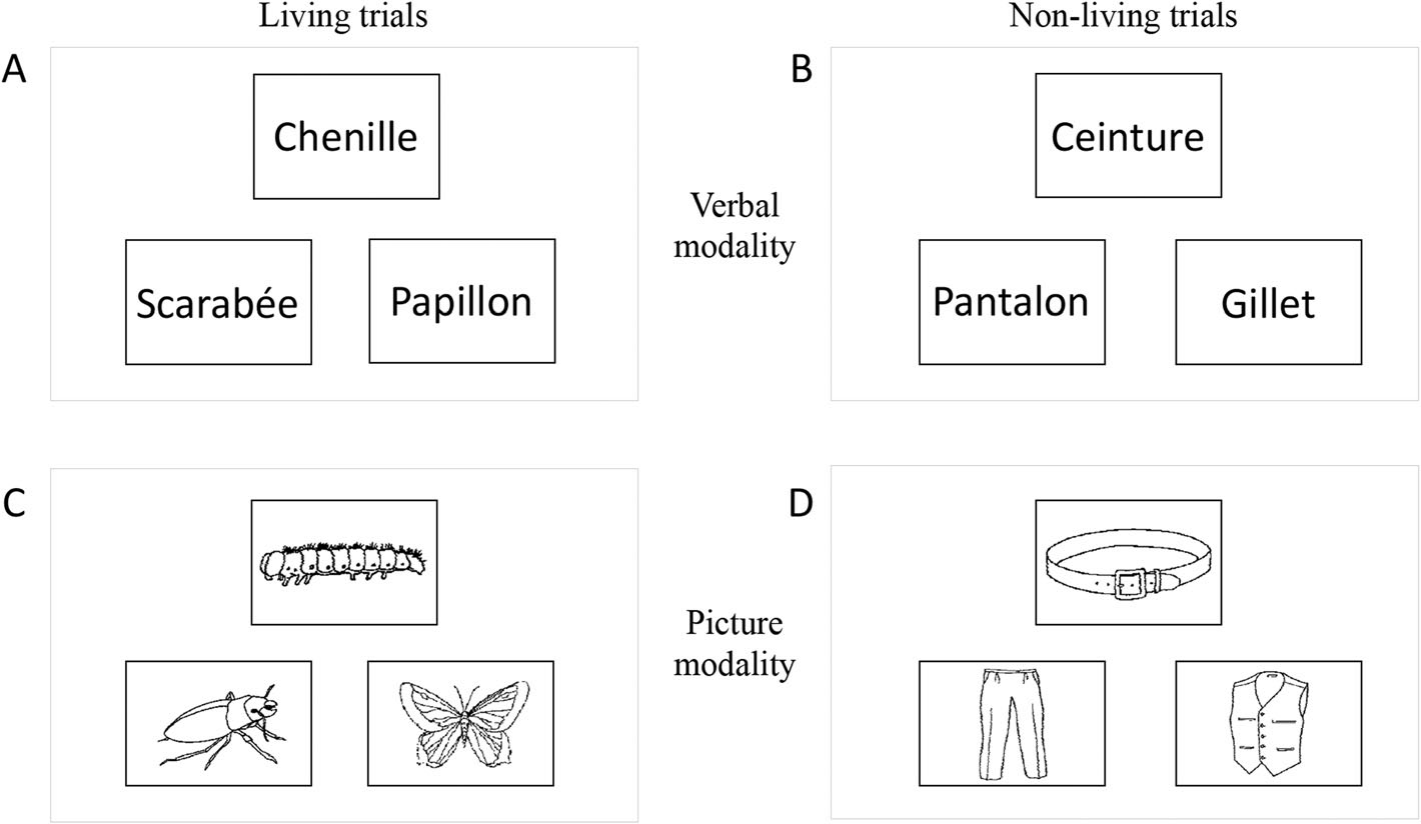
Two trials of the semantic association (SA) test in their verbal and picture modalities. **a** Trial with living items for the verbal modality (*chenille* (caterpillar) - target item, *scarabé*e (beetle) - distractor, papillon (butterfly) - test item). **b** Trial with living items for the picture modality using the same items in a picture format. **c** Trial with non-living items for the verbal modality (*ceinture* (belt) - target item, *pantalon* (trousers) - test item, *gilet* (vest) - distractor). **d** Trial with non-living items for the picture modality using the same items in a picture format. For each trial in each modality subjects have to decide, by pressing one of two buttons in the response box, which of the items presented on the bottom of the screen is associated with the target item, on the top of the screen

This task assesses semantic capacities and provides the primary endpoint criterion. It is based on the principle of the Pyramid Palm Trees Test [54]. The material includes 78 French words, which are grouped into 26 trials. Each trial includes three words, two of them are semantically related (the target item and test item), while the third word is a semantically unrelated distractor. The three words are displayed on the computer screen for 8 s and the participant has to decide, using two response buttons, if the target item (on top of the screen) is associated with the test item (on the bottom of the screen, either left or right) or the distractor item (on the bottom of the screen, either left or right, opposite to the test item). Thirteen test items appear on the left and 13 on the right. The lack of response during this 8-s time interval is recorded as an error. The task assesses two category dimensions using the contrast between trials containing only living items (*n* = 13) and trials containing only non-living items (*n* = 13). Two modalities of this task are applied: a verbal modality (described above) and a picture modality that uses pictures instead of words. The language stimuli (pictures or words) used in these two tasks for “living” and “non-living” trials are matched for (1) lexical frequency, (2) number of letters, (3) familiarity of words and pictures and (4) visual complexity of the pictures. Stimuli of version 1 and version 2 of the test are also matched for these four variables. Outcome measures will focus on performance (number of correct responses), and reaction times in milliseconds. Figure 3 illustrates different trials of the task.

#### Picture naming task (NAME)

This task evaluates lexical and semantic abilities. The material includes 40 pictures, derived from two picture-naming databases [55, 56]. Each picture is displayed on the computer screen for 8 s and participants are asked to name it aloud. Responses are voice-recorded and notified. The lack of a response during this time interval is recorded as an error. Stimuli of version 1 and version 2 of the test are matched for (1) lexical frequency of words, (2) familiarity of words and pictures and (3) visual complexity of the pictures. The task will allow for assessing (1) the number of correct responses, (2) the number of non-responses and (3) the number of semantic paraphasias. The number of correct responses and non-responses are markers of lexico-semantic abilities, and the number of semantic paraphasias is an additional marker of semantic abilities.

#### Reading task (READ)

The task provides a semantic marker in written language. During reading the phonological pathway allows for mapping each letter (grapheme) to a phoneme while the lexical-semantic pathway allows for “whole word reading” that depends on knowledge of written words [57]. The lexical-semantic route is therefore critical for reading irregular words where the grapheme-phoneme correspondence is not transparent (e.g., bear). In contrast, the phonological route is essential to read unknown or non-words. Regular words can be read through both the lexical-semantic and the phonological route. It has been shown that patients with SD have difficulties reading irregular words linked to their semantic impairment (e.g., see [58]) and performance with such irregular items will therefore provide a semantic marker. The task contains 45 stimuli: 15 irregular words, 15 regular words and 15 non-words that are matched for the number of graphemes. Each word appears on the computer screen for 8 s and subjects are asked to read them aloud. Responses are voice-recorded and registered in written form by the evaluator. The lack of response during this interval is recorded as an error. Stimuli of version 1 and version 2 of the test are also matched for the number of graphemes.

#### Verbal fluency task (FLU)

This task assesses language fluidity and access to lexical-semantic representations of words. It has two modalities: (1) in the “letter fluency” subtask participants are asked to produce, during 1 min, a maximum of words beginning with a particular letter displayed in the center of the screen; (2) in the “category fluency” subtask participants are asked to produce, during 1 min, a maximum of words belonging to a given semantic category displayed in the center of the computer screen. For the “letter fluency” subtask, stimuli of version 1 and version 2 are matched for the number of existing words starting with the given letters and also their cumulative frequencies. For the “category fluency” subtask stimuli of version 1 and version 2 are matched for the number of existing items within that category. The measured variables are the number of items produced per minute in each of the two tasks. The subjects’ responses are voice-recorded and quantified.

#### Category judgment task (CJ)

The task assesses semantic capacities in the verbal modality. The material includes 40 French words, 20 of which represent “living items” and 20 of which represent “non-living items”. Words representing “living” and “non-living” items, and words of both versions of the task, are matched for lexical frequency, number of letters and number of phonemes. Each word stimulus is displayed in the center of a computer screen for 8 s. Subjects are asked to judge whether a given word item belongs to a “living” or to a “non-living” semantic category and answer by pressing the corresponding buttons of the response box.

#### Executive function task (EXE)

**Fig. 4:**
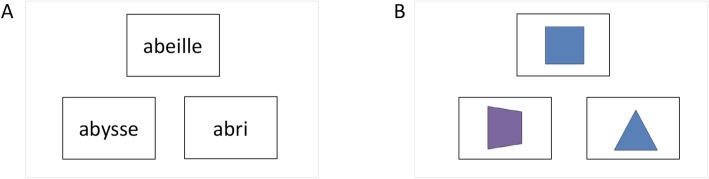
Two illustrative trials of the executive function (EXE) task. **a** Trial in the verbal modality. For each trial participants have to decide which of the test items presented on the bottom of the screen begins *and* ends with the same letter as the target item (*abeille* (bee) - target item, *abysse* (abyss) - test item, *abri* (shelter) - distractor). **b** Trial in the picture modality. For each trial subjects have to decide which of the test items on the bottom of the screen has the same color *or* the same geometric form as the target item

This task is used as a control task to assess whether tDCS over anterior temporal regions has semantic-specific effects or whether it might impact on executive functioning, which may indirectly modulate semantic performance. Using a similar task design and procedure as in the SA task, the EXE task assesses executive/attention and decision-making abilities without the influence of semantics. As in the SA task, the task contains a verbal and a picture modality. For the verbal modality, the material includes 78 French words grouped into 26 trials. Each trial includes three words; two of them have the same initial and final letters (the target item and test item), while the third word is a distractor sharing only the initial or the final letter with the target item. The three words presented in each trial are semantically unrelated. The picture modality of the test uses drawn images instead of words. The pictures represent colored geometrical shapes. The material includes 78 pictures grouped into 26 trials. Each of the 26 trials includes three pictures, two of them representing the same geometrical shape or color (the target item and test item), while the third picture represents a distractor not sharing any of these features with the target item. The items of the verbal modality are matched for the number of letters with the word items of the SA task. Stimuli of version 1 and version 2 are matched for the number of letters. The three items are displayed on the computer screen for 8 s and subjects decide (using two response buttons) if the target item (on top of the screen) is related to the test item (on the bottom of the screen, either left or right) or the distractor item (on the bottom of the screen, either left or right, opposite to the test item). Thirteen test items appear at a left and 13 at a right bottom location. The lack of response during this 8-s time interval is recorded as an error. Figure 4 illustrates two trials of this test.

#### Recognition of famous faces subtest

This test is adapted from the French BIP [59]. It is used to detect eventual negative effects of tDCS, decreasing the activity of the right ATL, which has been shown to play an important role in the recognition of known faces [7]. The material includes 28 pictures of faces of famous people. Each picture is displayed on the computer screen for 8 s. Participants have to decide if the face corresponds to one of the four professional categories (politician, actor, singer or TV presenter) by pressing one of four associated keys in the response box. The lack of response during this time interval is recorded as an error*.*

#### Ecological evaluation

Additionally, a semi-quantitative daily life communication questionnaire - *Echelle de Communication Verbale de Bordeaux* [60], which assesses the effectiveness of communication of patients with aphasia in everyday situations, is applied at baseline and at the 2-week post stimulation time point. This questionnaire was included in the protocol to count with an ecological measure of potential language improvements after tDCS and to assess its impact in patients’ day-to-day life.

### PET-MRI neuroimaging and EEG recording

Neuroimaging data (PET, MRI) are collected before the tDCS sessions (baseline) and 2 weeks after the tDCS sessions. These acquisitions provide all the sequences underlying the following explorations: cortical metabolism, gray matter thickness measures, white matter fiber tracking and functional connectivity. The examination is performed on a hybrid PET-MRI scanner (Signa 3 T GE Healthcare, USA). The injection of FDG (Fluoro-Deoxy-Glucose 2-18F: MÉTATRACE®, half-life of 109,77 min, or GLUSCAN®, half-life of 110 min) is performed if the blood glucose checked prior to injection is ≤ 1.5 g/l. Participants lie in neurosensory rest in a quiet and unlit room for at least 30 min post-injection, prior to image acquisition.

The acquisition of brain images with PET is conducted in *list mode*. It begins 30–40 min after injection of the radiopharmaceutical FDG tracer and lasts for 20 min (3 × 5 min). Images are reconstructed and corrected for physical phenomena. They are expressed in standard uptake value (SUV). Magnetic resonance images are acquired simultaneously and include two anatomical sequences (3D-T1, 3D-FLAIR), a functional imaging sequence (resting state fMRI) and a diffusion tensor imaging (DTI) sequence.

Additionally, prior to and following stimulation, the tDCS device (Startsim, NEuroelectrics, Barcelona) automatically records brain activity through EEG scalp electrodes (sampling at 1500 Hz, low-pass high-pass filter 4–40 Hz). Resting-state EEG recordings are obtained from 10 to 20 EEG system locations F4, F3, C4, C3, P4, P3 and T8, T7.

### Computational models of tDCS current magnitude and distribution

There is evidence that the thickness of the skull, the volume of the cerebrospinal fluid in the subdural space and the distance from the targeted region in the brain to the tDCS anode or cathode account for up to 50% of the spatial variation of the electric field strength [61–63]. Kim et al. [64] found that performance improvement in a working memory task correlated with the simulated current magnitude, suggesting that inconsistent behavioral outcomes of tDCS might be partly due to individual anatomical differences.

**Fig. 5:**
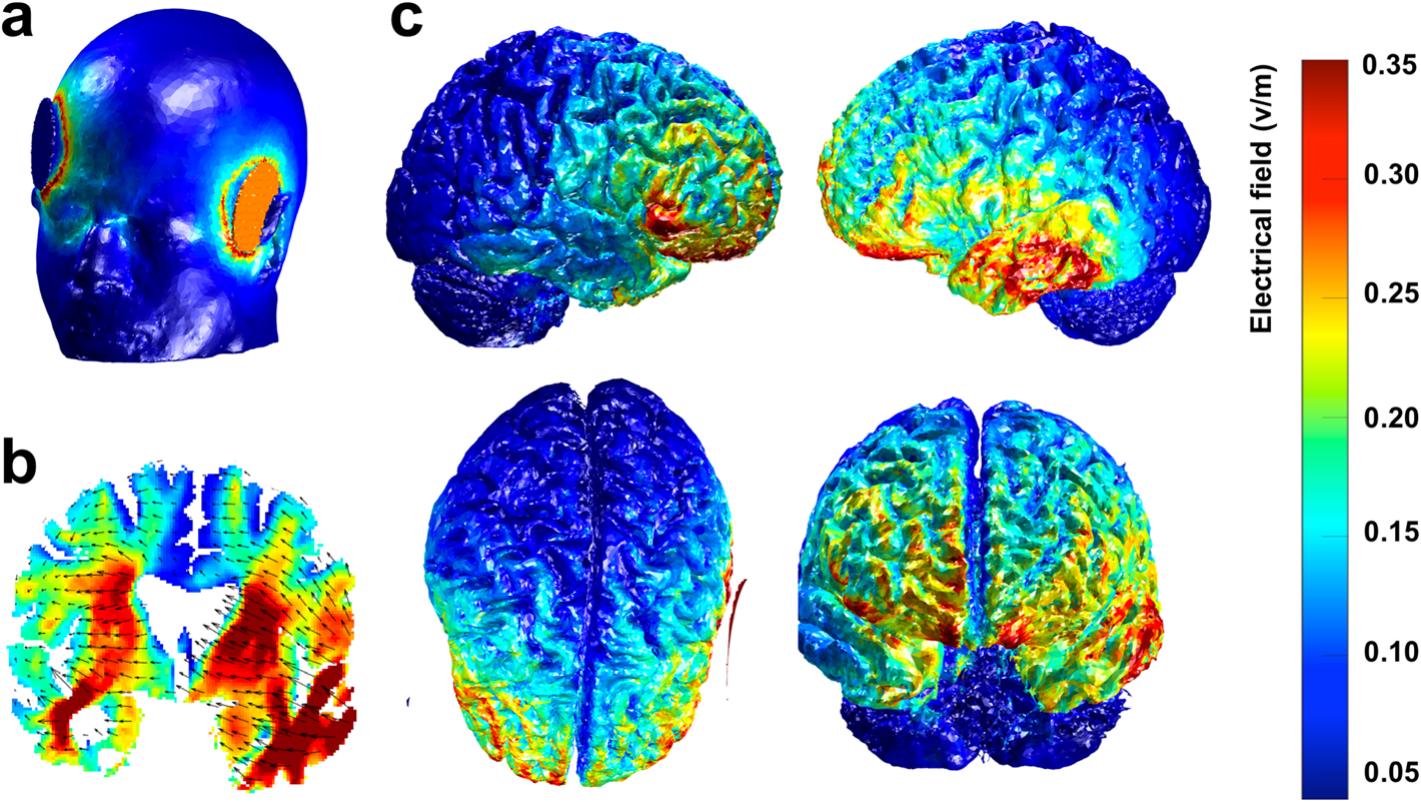
Example of a computational model produced with the open-source tool ROAST for the simulation of transcranial direct current stimulation (tDCS) current magnitude and distribution using a 3D T1-weighted image from a representative SD patient. **a** Illustration of the montage for the left-anodal stimulation. The round blue patch represents the cathode, placed over the right supra-orbital region [10–20 electroencephalography (EEG) coordinates AF8], and the red round patch represents the anode, placed over the left Anterior Temporal Lobe (ATL) (Montreal Neurological Institute (MNI) coordinates: x = − 52, y = 2, z = − 28). **b** Coronal slice view of the electric field with current flow direction represented by the black arrows. **c** Electric field distribution on the cortical surface - right hemisphere view, left hemisphere view, upper view and frontal view. The color-bar represents the magnitude of the electric field, in Volts per meter, in different regions of the brain (for panels **b** and **c**)

A computational approach of modeling should allow for defining if and how tDCS current magnitude and distribution in the heads of individuals can differ and how these differences will affect clinical outcomes. Individual computational models will be produced using the open-source tool ROAST [65] to simulate tDCS current magnitude and distribution using the anatomical 3D T1-weighted MRI images of each patient (Fig. 5). Then, correlation will be tested between model-estimated data for current magnitude values and clinical data, specifically the changes in scores in the SA task from baseline to post-stimulation. A region of interest (ROI) will be defined and the mean of the 5% highest electric field values in this region will be obtained for each patient. More specifically, we will define the left anterior/middle temporal lobe as our ROI, because this region is primarily damaged in SD and it is the region targeted during the stimulation. Finally, measures of thickness of the different tissue layers (skin, skull, cerebrospinal fluid volume, gray matter and white matter) and distances between the stimulation target (ATL) and the stimulating electrode will be obtained and regression models will be computed to identify specific features that most influence changes in current magnitudes. This will allow us to identify the characteristics that might influence patients’ responses to tDCS and help predict if an individual patient might benefit from tDCS treatment.

### Data management

Data are collected via the eCRF that was developed at the outset of the study. All clinical and language/semantic task information required by the protocol is entered into the eCRF. The data are collected as and when they are obtained, and any missing data are clearly coded. Every investigator participating in the protocol has access to the eCRF via a web-based password-protected data collection system. All data are collected in the same center, the Pitié-Salpêtrière Hospital. Investigators have been given instructions for using this tool prior to the beginning of the protocol. Regular monitoring by the promoter of the study (APHP. Assistace Publique-Hôpitaux de Paris) will ensure the accuracy and quality of all the data and detect and address any issues related to the implementation.

All data collected on participants are anonymized through a specific number attributed to the participant at inclusion and his/her respective last and first name initials. Data collected in this study include both quantitative (neuropsychological and language standard tests scores, study task scores) and qualitative data (clinical history, neurological information). A print-out, authenticated by the principal investigator of the protocol, will be requested at the end of the research by the promoter of the study and the investigator will archive a copy of the authenticated document that was issued to the promoter. Given the high volume and complexity of the pre-processing and analyses of PET-MRI and EEG data, the outcome measures cannot be added to the eCRF and will be handled by the expert investigators.

### Data analyses

We will accept a significance level of 5% (*p* < 0.05) corrected for multiple comparisons when needed. To assess our primary endpoint criterion, i.e., performance changes on the SA task, outcomes at baseline and at the 2-week time point will be compared using two-way analysis of variance with “time” (baseline, 2 weeks post-stimulation) and “group” (anodal, cathodal, sham) as factors. Since two comparisons will be made (sham versus left-anodal and sham versus right-cathodal tDCS) Dunnett’s two-tailed *t* test will be used to handle multiple comparisons. In the case that the data do not meet the normal distribution assumption, the non-parametric Mann-Whitney test will be used to compare performance improvements between the sham versus left-anodal and between the sham versus right-cathodal groups.

To assess the time course of potential improvements and test if such differ between the treatment groups, linear mixed models will be used with “time” (the four time points), “group” (anodal, cathodal and sham) and group-by-time interactions as fixed effects. The Benjamini and Hochberg method will be used for controlling for false discovery rate.

To identify imaging bio-markers of stimulation-induced neuroplasticity, we will analyze cortical metabolism and resting-state functional connectivity. PET images will be used for cortical metabolism, to analyze tissue metabolic activity and regional glucose uptake in the brain. A voxel-based analysis, using the Statistical Parametric Mapping (SPM) software [66], will be implemented to highlight regional differences in metabolism between the images obtained at baseline and post-stimulation. The resting-state fMRI sequence will be explored to study the status of functional connectivity within the language/semantic networks and possible alterations within it between baseline and post-stimulation images. Quantification of functional connectivity will be based on integration measures in specific networks established from the known anatomy, on indices derived from the graph theory or information theory. Cortical regions of interest will be obtained automatically using SPM toolboxes. More specifically, the language/semantic network will include the middle and anterior portions of the lateral ATL, the inferior frontal gyrus, the dorsomedial and ventromedial prefrontal cortex and the inferior parietal lobe. To study the neurophysiological impact of tDCS on specific brain networks, resting-state EEG datasets obtained at baseline and 3 days after the end of the 10 tDCS sessions will be also analyzed. Data will be filtered (4–40 Hz) and power distribution and local synchrony calculated across frequency bands and compared. Functional connectivity maps across the electrodes will be estimated by calculating the phase-lockimg value between the eight EEG leads and have them compared across in pairs.

To identify biomarkers that could individually predict an efficient tDCS impact, 3D-T1 and DTI sequences will be explored and individual computational models of tDCS current will be produced. Structural 3D-T1 MRI data will be studied using surface-based cortical thickness analysis. An ROI will be defined in the ATL and cortical thickness values at baseline between a group of eventual “responders” (mean improvement of 15% in correct responses in the SA test) and “non-responders” to the stimulation will be compared using a general linear model. Diffusion MRI data will be studied using ROI analysis of DTI metrics. Fractional anisotropy (FA) and mean diffusivity (MD) maps will be calculated and the integrity of a set of anatomical white matter tracts will be assessed (FA and MD measures in each tract). The tracts analyzed will include bilaterally the inferior longitudinal fasciculus, the uncinate fasciculus, the superior longitudinal fasciculus, the inferior frontal-occipital fasciculus and different corpus callosum tracts connecting the left and right temporal lobes. This will allow a detailed anatomical analysis of white matter fiber tracts and compare their status between a possible group of responders and non-responders. The individual computational models of tDCS current magnitude will be used to identify which anatomical characteristics influence the amount of current reaching the target in the brain and if such measure can help predict the response to stimulation. Finally, the analyses of DTI data will also allow us to explore connectivity between the left and right ATL to improve the understanding of the semantic roles of each of these regions while contributing to the definition of anatomic-functional models of semantics.

### Sample size

The primary evaluation criterion is the change over 2 weeks in performance on the SA task. Performance is measured by the percentage of correct responses. The trial will be considered as positive if performances after left-anodal or right-cathodal tDCS are shown to be significantly superior to sham stimulation. The sample size is based on the results of a preliminary study comparing sham stimulation to left-anodal and right-cathodal stimulation in 12 patients [45]. In our pre-therapeutic study, the mean difference in performance improvement between sham and both anodal and cathodal stimulation was about 15% with a standard deviation of 16% and an effect size of 1.464. The difference in mean change between sham stimulation and each of the two other groups is then expected to be 15% of correct responses. The inclusion of 20 patients in each group will provide statistical power of 80%.

## Discussion

Language disorders in frontotemporal lobar degeneration, and particularly in SD, are a disabling feature representing an important medical problem. They are also a relevant issue for public health because most patients have symptoms prior to retirement causing substantial health costs. Given this context our project bears major importance because it could potentially provide evidence for the validity of a novel therapy strategy improving language/semantic capacities while diminishing the functional handicap and, eventually, healthcare expenditure.

The STIM-SD protocol proposes the first large-scale exploration of tDCS as a potential therapy for language/semantic impairment in SD for which no treatment is currently available. Contrary to most of the studies using transcranial brain stimulation in neurodegenerative diseases affecting language [43, 44, 67, 68], our study targets sites that have been selected based on the localization of anatomical damage and related contralateral regions to optimize language and semantic recovery.

We apply a double-blind, sham-controlled design in which the investigators and the patients are blinded to the type of stimulation used, reducing any source of potential bias. This design will also enable a comparison between two different stimulation approaches (left-anodal versus right-cathodal) and will contribute to identifying the most beneficial strategy. To our knowledge, not a single study applying tDCS to improve cognitive deficits in neurodegenerative diseases has employed supportive neuroimaging (PET, fMRI), and only one study has used neurophysiological measures (EEG) [69] to explore stimulation impact on relevant brain networks or demonstrate neuroplasticity effects. In the present study, we will generate both language/semantic data and a set of neuroimaging and resting-state EEG measures, which will allow us to study the impact of tDCS on language networks and neuroplasticity, to understand if this potential impact also subtends language/semantic improvements, and to better understand tDCS mechanisms.

In addition, baseline structural data (3D-T1 cortical thickness, DTI fiber tract analyses) and individualized models of tDCS current magnitude and distribution will be fundamental in understanding which anatomical features of our cohort most influence treatment efficacy, how the amount and spreading of electric current in the brain will impact individual clinical outcomes and possibly define a profile of patients that in the future would most benefit from tDCS.

Within neurodegenerative diseases specifically affecting language, SD is the most frequent variant of primary progressive aphasia [2], which contributes to the feasibility of the project in terms of patient recruitment. In the same vein, our National Reference Center for Rare or Early Onset Dementias provides a unique opportunity to recruit a large and homogeneous cohort of patients with SD. The active patient files on patients with early-stage SD in the three recruiting centers contained more than 20 patients with SD per year in 2013, 2014 and 2015. This shows that the total number of 60 patients with SD can be reached in the current project.

A potential tDCS treatment would be easily applicable, inexpensive and renewable when therapeutic effects disappear due to disease evolution. Significant effects of repetitive tDCS on language/semantics would also open an avenue for future tDCS trials targeting language non-related cortical regions such as areas subtending episodic memory, which is damaged, for example, in Alzheimer’s disease. More generally, the protocol might improve the understanding of neuroplasticity and its modulation through inhibitory and/or excitatory tDCS-driven cortical impact, while providing a rationale for appropriate stimulation modalities and for identifying brain regions likely to demonstrate relevant plasticity. Such insights could prove important for both tDCS and TMS trials, and their combination with behavioral language rehabilitation strategies, which might further enhance plasticity-related modulation.

## Conclusions

The aim of the STIM-SD protocol is to implement a novel therapeutic tDCS approach to language/semantic deficits in patients with SD for whom no treatment is available. If found to be efficient, this strategy could be regularly implemented, as it is easily applicable and low-cost. Moreover, larger trials could be extended to other neurodegenerative diseases to check for efficiency in language and other cognitive functions.

### Trial status

The protocol version number is P160937J first published in ClinicalTrials.gov on 29 March 2018. Patient recruitment began in June 2018. Sixteen patients have been screened for participation between June 2018 and March 2019. Of these, 13 patients were included in the study and randomized. Seven of those patients have already completed their participation in the study with 4-month follow up. Among the three patients screened but not included, two patients did not meet all the inclusion criteria while another was not able to undergo the planned neuroimaging examinations. Five healthy participants were also included in the protocol. Recruitment is expected to be completed by June 2021.

## Additional file

Additional file 1: Standard protocol items: recommendation for interventional trials (SPIRIT) 2013 checklist: recommended items to address in a clinical trial protocol and related documents. (DOC 122 kb)

## Data Availability

Not applicable. No data is available at this point because authors are still in the process of gathering such.
